# Tris[2-meth­oxy-6-(4-methyl­phenyl­iminio­meth­yl)phenolato-κ^2^ *O*,*O*′]tris­(thio­cyanato-κ*N*)neodymium(III)

**DOI:** 10.1107/S1600536809053124

**Published:** 2009-12-16

**Authors:** Jia-Lu Liu, Jin-Bei Shen, Guo-Liang Zhao

**Affiliations:** a Zhejiang Key Laboratory for Reactive Chemistry on Solid Surfaces, Institute of Physical Chemistry, Zhejiang Normal University, Jinhua, Zhejiang 321004, People’s Republic of China, and College of Chemistry and Life Science, Zhejiang Normal University, Jinhua 321004, Zhejiang, People’s Republic of China

## Abstract

In the title compound, [Nd(NCS)_3_(C_15_H_15_NO_2_)_3_], the Nd^III^ ion is coordinated by three thio­cyanate anions [Nd—N = 2.489 (8)–2.530 (7) Å] and six O atoms [Nd—O = 2.375 (4)–2.843 (5) Å] from three zwitterionic 2-meth­oxy-6-(4-methyl­phenyl­iminiometh­yl)phenolate ligands in a tricapped trigonal-prismatic geometry. Intra­molecular N—H⋯O hydrogen bonds occur. The crystal packing exhibits weak inter­molecular C—H⋯S hydrogen bonds, π–π inter­actions with a distance of 3.904 (7) Å between the centroids of the aromatic rings, and voids of 101 Å^3^.

## Related literature

For related structures, see: Wang & Chang (1994 ▶); Zhao *et al.* (2007 ▶); Xian *et al.* (2008 ▶); Li *et al.* (2008 ▶).
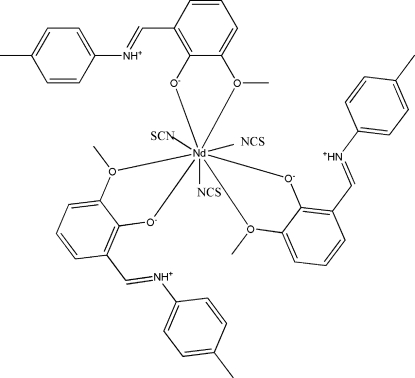


## Experimental

### 

#### Crystal data

[Nd(NCS)_3_(C_15_H_15_NO_2_)_3_]
*M* *_r_* = 1042.35
Monoclinic, 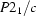

*a* = 16.6107 (3) Å
*b* = 14.2425 (3) Å
*c* = 22.1582 (4) Å
β = 105.972 (1)°
*V* = 5039.77 (17) Å^3^
*Z* = 4
Mo *K*α radiation
μ = 1.21 mm^−1^
*T* = 296 K
0.14 × 0.08 × 0.05 mm

#### Data collection

Bruker APEXII diffractometer
44769 measured reflections
11604 independent reflections
5189 reflections with *I* > 2σ(*I*)
*R*_int_ = 0.114

#### Refinement

*R*[*F* ^2^ > 2σ(*F* ^2^)] = 0.060
*wR*(*F* ^2^) = 0.190
*S* = 0.86
11604 reflections
562 parameters
H-atom parameters constrained
Δρ_max_ = 0.66 e Å^−3^
Δρ_min_ = −0.84 e Å^−3^

### 

Data collection: *APEX2* (Bruker, 2006 ▶); cell refinement: *SAINT* (Bruker, 2006 ▶); data reduction: *SAINT*; program(s) used to solve structure: *SHELXS97* (Sheldrick, 2008 ▶); program(s) used to refine structure: *SHELXL97* (Sheldrick, 2008 ▶); molecular graphics: *SHELXTL* (Sheldrick, 2008 ▶); software used to prepare material for publication: *SHELXL97*.

## Supplementary Material

Crystal structure: contains datablocks I, global. DOI: 10.1107/S1600536809053124/cv2653sup1.cif

Structure factors: contains datablocks I. DOI: 10.1107/S1600536809053124/cv2653Isup2.hkl

Additional supplementary materials:  crystallographic information; 3D view; checkCIF report

## Figures and Tables

**Table 1:** Hydrogen-bond geometry (Å, °)

*D*—H⋯*A*	*D*—H	H⋯*A*	*D*⋯*A*	*D*—H⋯*A*
N1—H1*A*⋯O2	0.86	1.86	2.566 (7)	138
N2—H2*A*⋯O4	0.86	1.87	2.576 (6)	138
N3—H3*A*⋯O6	0.86	1.89	2.589 (7)	137
C38—H38*A*⋯S1^i^	0.93	2.72	3.633 (6)	166

Symmetry code: (i) 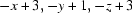.

**Fig. 1.**
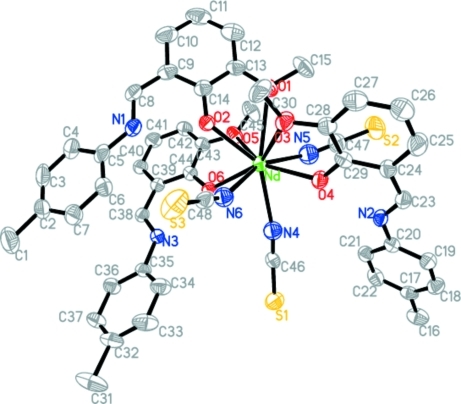
The molecular structure of the title complex, showing the atom-labelling scheme. Displacement ellipsoids are drawn at the 30% probability level. H atoms omitted for clarity.

## supplementary crystallographic information

### Comment

Schiff base ligands derived from substituted salicylaldehyde and their metal complexes have been widely investigated because of their structural features (Wang *et al.*,1994). In this aspect we have been synthesized several analogous Schiff bases transitional and rare metal complexes. In recent articles we have reported our research results (Zhao *et al.*, 2007; Xian *et al.*, 2008; Li *et al.*, 2008). Herein, we report the crystal structure of the title Nd^3+^ complex, (I).

In (I) (Fig. 1), the Nd^III^ is nine- coordinated by three nitrogen atoms from three thiocyanate ions and six O atoms from 2-(4-methylphenyliminomethyl)-6-methoxyphenolato) (*L*). *L* ligands coordinate the Nd center with bidentate-chelate mode using oxygen atoms from deprotonated phenolic hydroxyl groups and methoxyl groups. The imino H atoms in  *L* are involved in intramolecular N—H···O hydrogen bonds (Table 1). The bonds between Nd^III^ and O atoms from phenolic hydroxyl groups are 2.375 (2) -2.393 (3) Å, which are shorter than those between Nd and O atoms of methoxyl groups (2.742 (3) - 2.843 (3) Å). The Nd—N bond lengths are 2.489 (4) -2.530 (4) Å. The structure of (I) is similar to the structures of analogous complexes (Zhao *et al.*, 2007; Li *et al.*, 2008).

The crystal packing exhibits weak intermolecular C—H···S hydrogen bonds  (Table 1) and π-π interaction with the distance of 3.904 (7) Å between the centroids of aromatic rings.

### Experimental

Reagents and solvents were of commercially available quality and were used without further purification. The Schiff base ligand was prepare in a high yield synthesis by condensation of *o*-vanillin and *p*-methyllaniline and was recrystallized in ethanol before being used. 1 mmol N d(NO^3^)^3^ (dissolved in methanol) was added dropwise into a methanol solution with 3 mmol 3-methoxy-*N*-salicylidene-*p*-toluidine under stirring and the mixture was continuously stirred at room temperature for 8 h to obtain a red solution. The deposit was filtered off and the solution was left standing for slow evaporation. Red crystals of the title compound were obtained after several days.

### Refinement

All H atoms were positioned geometrically (C—H 0.93–0.96 Å, N—H 0.86 Å), and refined as riding, with *U*_iso_(H) = 1.2–1.5*U*_eq_(C, N). The crystal packing exhibits voids of 101 Å^3^ centered at (0.19, 0.67, 0.24).

### Crystal data

**Table:**

[Nd(NCS)_3_(C_15_H_15_NO_2_)_3_]	*F*(000) = 2124
*M**_r_* = 1042.35	*D*_x_ = 1.374 Mg m^−^^3^
Monoclinic, *P*2_1_/*c*	Mo *K*α radiation, λ = 0.71073 Å
Hall symbol: -P 2ybc	Cell parameters from 2091 reflections
*a* = 16.6107 (3) Å	θ = 1.4–27.6°
*b* = 14.2425 (3) Å	µ = 1.21 mm^−^^1^
*c* = 22.1582 (4) Å	*T* = 296 K
β = 105.972 (1)°	Block, red
*V* = 5039.77 (17) Å^3^	0.14 × 0.08 × 0.05 mm
*Z* = 4	

### Data collection

**Table:**

Bruker APEXII diffractometer	5189 reflections with *I* > 2σ(*I*)
Radiation source: fine-focus sealed tube	*R*_int_ = 0.114
graphite	θ_max_ = 27.6°, θ_min_ = 1.3°
ω scans	*h* = −21→21
44769 measured reflections	*k* = −18→18
11604 independent reflections	*l* = −28→28

### Refinement

**Table:**

Refinement on *F*^2^	Primary atom site location: structure-invariant direct methods
Least-squares matrix: full	Secondary atom site location: difference Fourier map
*R*[*F*^2^ > 2σ(*F*^2^)] = 0.060	Hydrogen site location: inferred from neighbouring sites
*wR*(*F*^2^) = 0.190	H-atom parameters constrained
*S* = 0.86	*w* = 1/[σ^2^(*F*_o_^2^) + (0.1*P*)^2^] where *P* = (*F*_o_^2^ + 2*F*_c_^2^)/3
11604 reflections	(Δ/σ)_max_ = 0.001
562 parameters	Δρ_max_ = 0.66 e Å^−^^3^
0 restraints	Δρ_min_ = −0.84 e Å^−^^3^

### Special details

**Table:**

Geometry. All e.s.d.'s (except the e.s.d. in the dihedral angle between two l.s. planes) are estimated using the full covariance matrix. The cell e.s.d.'s are taken into account individually in the estimation of e.s.d.'s in distances, angles and torsion angles; correlations between e.s.d.'s in cell parameters are only used when they are defined by crystal symmetry. An approximate (isotropic) treatment of cell e.s.d.'s is used for estimating e.s.d.'s involving l.s. planes.
Refinement. Refinement of *F*^2^ against ALL reflections. The weighted *R*-factor *wR* and goodness of fit *S* are based on *F*^2^, conventional *R*-factors *R* are based on *F*, with *F* set to zero for negative *F*^2^. The threshold expression of *F*^2^ > σ(*F*^2^) is used only for calculating *R*-factors(gt) *etc*. and is not relevant to the choice of reflections for refinement. *R*-factors based on *F*^2^ are statistically about twice as large as those based on *F*, and *R*- factors based on ALL data will be even larger.

### Fractional atomic coordinates and isotropic or equivalent isotropic displacement parameters (Å^2^)

**Table:**

	*x*	*y*	*z*	*U*_iso_*/*U*_eq_	
Nd	1.21267 (2)	0.27661 (3)	1.393189 (17)	0.04942 (15)	
C1	1.7474 (6)	0.2005 (8)	1.6278 (6)	0.132 (4)	
H1B	1.7970	0.1935	1.6140	0.197*	
H1C	1.7517	0.1616	1.6639	0.197*	
H1D	1.7416	0.2649	1.6386	0.197*	
C2	1.6709 (6)	0.1709 (6)	1.5751 (5)	0.090 (3)	
C3	1.6775 (5)	0.1410 (7)	1.5180 (6)	0.098 (3)	
H3B	1.7303	0.1368	1.5115	0.118*	
C4	1.6086 (5)	0.1167 (6)	1.4699 (4)	0.079 (2)	
H4A	1.6150	0.0969	1.4315	0.095*	
C5	1.5300 (5)	0.1220 (5)	1.4793 (4)	0.064 (2)	
C6	1.5214 (5)	0.1515 (5)	1.5365 (4)	0.066 (2)	
H6A	1.4686	0.1552	1.5430	0.079*	
C7	1.5906 (6)	0.1754 (6)	1.5834 (4)	0.082 (2)	
H7A	1.5842	0.1951	1.6218	0.099*	
C8	1.4479 (5)	0.0514 (5)	1.3821 (4)	0.065 (2)	
H8A	1.4939	0.0182	1.3772	0.078*	
C9	1.3712 (5)	0.0448 (5)	1.3347 (4)	0.069 (2)	
C10	1.3636 (7)	−0.0112 (6)	1.2797 (5)	0.097 (3)	
H10A	1.4097	−0.0448	1.2752	0.116*	
C11	1.2900 (8)	−0.0158 (8)	1.2340 (5)	0.110 (4)	
H11A	1.2860	−0.0531	1.1988	0.132*	
C12	1.2212 (7)	0.0342 (7)	1.2392 (4)	0.096 (3)	
H12A	1.1713	0.0307	1.2073	0.115*	
C13	1.2253 (5)	0.0892 (5)	1.2909 (4)	0.066 (2)	
C14	1.3004 (5)	0.0943 (5)	1.3399 (4)	0.0613 (19)	
C15	1.0798 (5)	0.1339 (7)	1.2583 (4)	0.098 (3)	
H15A	1.0791	0.0822	1.2304	0.147*	
H15B	1.0642	0.1904	1.2344	0.147*	
H15C	1.0408	0.1221	1.2823	0.147*	
C16	0.9337 (6)	0.8193 (6)	1.4168 (4)	0.092 (3)	
H16A	0.9816	0.8487	1.4085	0.138*	
H16B	0.9289	0.8393	1.4570	0.138*	
H16C	0.8841	0.8368	1.3848	0.138*	
C17	0.9441 (5)	0.7145 (5)	1.4168 (3)	0.0619 (19)	
C18	0.8840 (5)	0.6555 (6)	1.4276 (4)	0.069 (2)	
H18A	0.8360	0.6809	1.4348	0.083*	
C19	0.8936 (4)	0.5584 (5)	1.4279 (3)	0.0601 (19)	
H19A	0.8524	0.5195	1.4354	0.072*	
C20	0.9633 (4)	0.5209 (5)	1.4171 (3)	0.0523 (17)	
C21	1.0259 (4)	0.5778 (5)	1.4075 (3)	0.0604 (19)	
H21A	1.0746	0.5520	1.4018	0.072*	
C22	1.0147 (5)	0.6738 (5)	1.4067 (4)	0.071 (2)	
H22A	1.0560	0.7124	1.3991	0.085*	
C23	0.9246 (4)	0.3560 (5)	1.4198 (3)	0.0612 (19)	
H23A	0.8711	0.3726	1.4217	0.073*	
C24	0.9442 (4)	0.2599 (5)	1.4191 (3)	0.0576 (19)	
C25	0.8829 (5)	0.1900 (6)	1.4185 (5)	0.089 (3)	
H25A	0.8296	0.2076	1.4200	0.107*	
C26	0.9016 (6)	0.0991 (7)	1.4158 (5)	0.105 (3)	
H26A	0.8606	0.0539	1.4142	0.126*	
C27	0.9813 (5)	0.0716 (6)	1.4151 (4)	0.083 (3)	
H27A	0.9926	0.0079	1.4132	0.099*	
C28	1.0438 (4)	0.1343 (5)	1.4174 (4)	0.0600 (19)	
C29	1.0261 (4)	0.2302 (5)	1.4194 (3)	0.0495 (16)	
C30	1.1531 (5)	0.0226 (5)	1.4252 (4)	0.086 (3)	
H30A	1.1104	−0.0169	1.4333	0.129*	
H30B	1.2027	0.0188	1.4598	0.129*	
H30C	1.1656	0.0022	1.3875	0.129*	
C31	1.6013 (7)	0.4363 (7)	1.7675 (4)	0.122 (4)	
H31A	1.5565	0.4274	1.7866	0.183*	
H31B	1.6238	0.4984	1.7765	0.183*	
H31C	1.6446	0.3910	1.7840	0.183*	
C32	1.5683 (6)	0.4236 (6)	1.6970 (4)	0.079 (2)	
C33	1.4863 (6)	0.3989 (6)	1.6687 (4)	0.085 (3)	
H33A	1.4496	0.3904	1.6933	0.102*	
C34	1.4576 (5)	0.3865 (6)	1.6047 (4)	0.073 (2)	
H34A	1.4018	0.3714	1.5862	0.088*	
C35	1.5109 (4)	0.3963 (5)	1.5688 (3)	0.0514 (17)	
C36	1.5935 (4)	0.4198 (5)	1.5956 (4)	0.063 (2)	
H36A	1.6301	0.4266	1.5707	0.075*	
C37	1.6216 (5)	0.4334 (6)	1.6600 (4)	0.075 (2)	
H37A	1.6773	0.4493	1.6783	0.090*	
C38	1.5238 (4)	0.3633 (4)	1.4624 (3)	0.0523 (17)	
H38A	1.5819	0.3674	1.4765	0.063*	
C39	1.4875 (4)	0.3426 (4)	1.3990 (3)	0.0482 (16)	
C40	1.5387 (4)	0.3304 (5)	1.3584 (4)	0.064 (2)	
H40A	1.5964	0.3373	1.3739	0.077*	
C41	1.5055 (5)	0.3089 (6)	1.2974 (4)	0.072 (2)	
H41A	1.5403	0.3005	1.2714	0.087*	
C42	1.4185 (5)	0.2993 (5)	1.2729 (3)	0.0610 (19)	
H42A	1.3953	0.2865	1.2305	0.073*	
C43	1.3680 (4)	0.3090 (5)	1.3120 (3)	0.0522 (17)	
C44	1.4000 (4)	0.3316 (5)	1.3758 (3)	0.0483 (16)	
C45	1.2416 (5)	0.3000 (7)	1.2278 (4)	0.091 (3)	
H45A	1.2821	0.3112	1.2050	0.137*	
H45B	1.1993	0.3478	1.2178	0.137*	
H45C	1.2162	0.2397	1.2166	0.137*	
C46	1.2340 (4)	0.5040 (5)	1.4736 (3)	0.0591 (19)	
C47	1.0439 (6)	0.3648 (7)	1.2736 (4)	0.090 (3)	
C48	1.3000 (4)	0.1762 (5)	1.5462 (3)	0.0577 (18)	
N1	1.4566 (4)	0.1018 (4)	1.4318 (3)	0.0628 (16)	
H1A	1.4113	0.1262	1.4365	0.075*	
N2	0.9766 (3)	0.4219 (4)	1.4181 (2)	0.0510 (14)	
H2A	1.0258	0.4041	1.4175	0.061*	
N3	1.4809 (3)	0.3773 (4)	1.5032 (3)	0.0499 (14)	
H3A	1.4274	0.3748	1.4883	0.060*	
N4	1.2184 (4)	0.4345 (5)	1.4465 (3)	0.0754 (18)*	
N5	1.1129 (5)	0.3612 (5)	1.3056 (4)	0.094 (2)*	
N6	1.2775 (4)	0.2273 (5)	1.5051 (3)	0.0677 (17)*	
S1	1.25218 (13)	0.60404 (18)	1.51067 (13)	0.0996 (9)	
S2	0.94976 (19)	0.3689 (2)	1.23046 (17)	0.1467 (14)	
S3	1.3324 (2)	0.1029 (2)	1.60350 (12)	0.1132 (10)	
O1	1.1632 (3)	0.1444 (4)	1.3004 (2)	0.0752 (15)	
O2	1.3015 (3)	0.1459 (3)	1.3889 (2)	0.0561 (12)	
O3	1.1243 (3)	0.1167 (3)	1.4178 (2)	0.0674 (14)	
O4	1.0848 (3)	0.2913 (3)	1.4208 (2)	0.0595 (13)	
O5	1.2824 (3)	0.3020 (4)	1.2938 (2)	0.0641 (13)	
O6	1.3500 (2)	0.3412 (3)	1.4114 (2)	0.0538 (12)	

### Atomic displacement parameters (Å^2^)

**Table:**

	*U*^11^	*U*^22^	*U*^33^	*U*^12^	*U*^13^	*U*^23^
Nd	0.0425 (2)	0.0534 (3)	0.0489 (3)	0.00325 (17)	0.00670 (16)	0.0035 (2)
C1	0.084 (7)	0.122 (9)	0.152 (11)	0.005 (6)	−0.028 (7)	0.001 (8)
C2	0.073 (6)	0.069 (6)	0.104 (8)	0.013 (5)	−0.016 (6)	−0.005 (6)
C3	0.053 (5)	0.092 (7)	0.134 (10)	0.022 (5)	0.000 (6)	0.007 (7)
C4	0.066 (5)	0.084 (6)	0.088 (7)	0.018 (4)	0.019 (5)	0.007 (5)
C5	0.072 (5)	0.040 (4)	0.078 (6)	0.018 (4)	0.016 (5)	0.015 (4)
C6	0.061 (5)	0.068 (5)	0.063 (5)	0.001 (4)	0.009 (4)	0.007 (4)
C7	0.091 (6)	0.059 (5)	0.083 (7)	0.012 (5)	0.001 (5)	0.010 (5)
C8	0.074 (5)	0.048 (5)	0.073 (6)	0.015 (4)	0.023 (5)	0.001 (4)
C9	0.092 (6)	0.062 (5)	0.058 (5)	0.003 (4)	0.029 (5)	−0.010 (4)
C10	0.116 (8)	0.086 (7)	0.099 (8)	0.001 (6)	0.045 (7)	−0.031 (6)
C11	0.140 (10)	0.117 (9)	0.074 (7)	−0.021 (8)	0.031 (7)	−0.051 (6)
C12	0.118 (8)	0.094 (7)	0.073 (7)	−0.016 (6)	0.023 (6)	−0.016 (6)
C13	0.084 (6)	0.059 (5)	0.050 (5)	−0.002 (4)	0.013 (4)	−0.005 (4)
C14	0.076 (5)	0.050 (4)	0.057 (5)	0.002 (4)	0.018 (4)	0.004 (4)
C15	0.079 (6)	0.099 (7)	0.086 (7)	−0.020 (5)	−0.029 (5)	0.007 (6)
C16	0.122 (7)	0.054 (5)	0.085 (7)	0.000 (5)	0.003 (6)	0.002 (5)
C17	0.078 (5)	0.047 (5)	0.049 (5)	0.005 (4)	−0.003 (4)	−0.002 (4)
C18	0.074 (5)	0.065 (5)	0.067 (5)	0.011 (4)	0.019 (4)	−0.001 (4)
C19	0.062 (4)	0.055 (5)	0.065 (5)	−0.003 (3)	0.021 (4)	0.002 (4)
C20	0.055 (4)	0.052 (4)	0.049 (4)	−0.006 (3)	0.012 (3)	−0.001 (3)
C21	0.056 (4)	0.054 (5)	0.066 (5)	0.001 (4)	0.009 (4)	0.000 (4)
C22	0.063 (5)	0.057 (5)	0.083 (6)	−0.006 (4)	0.003 (4)	0.011 (5)
C23	0.052 (4)	0.060 (5)	0.075 (5)	−0.001 (4)	0.023 (4)	−0.006 (4)
C24	0.053 (4)	0.063 (5)	0.059 (5)	−0.004 (3)	0.018 (4)	−0.002 (4)
C25	0.076 (5)	0.072 (6)	0.136 (9)	−0.014 (5)	0.056 (6)	−0.012 (6)
C26	0.091 (7)	0.070 (7)	0.161 (10)	−0.026 (5)	0.046 (7)	−0.023 (7)
C27	0.096 (6)	0.047 (5)	0.104 (7)	−0.007 (5)	0.027 (6)	−0.002 (5)
C28	0.063 (4)	0.036 (4)	0.079 (6)	−0.004 (3)	0.017 (4)	0.003 (4)
C29	0.056 (4)	0.051 (4)	0.042 (4)	−0.012 (3)	0.013 (3)	0.003 (3)
C30	0.098 (6)	0.042 (5)	0.128 (8)	0.020 (4)	0.045 (6)	0.014 (5)
C31	0.189 (11)	0.114 (9)	0.051 (6)	−0.040 (8)	0.012 (7)	−0.017 (6)
C32	0.111 (7)	0.070 (6)	0.049 (5)	−0.015 (5)	0.013 (5)	−0.015 (4)
C33	0.112 (7)	0.090 (7)	0.064 (6)	−0.031 (5)	0.042 (6)	−0.018 (5)
C34	0.074 (5)	0.088 (6)	0.062 (6)	−0.017 (4)	0.026 (5)	−0.017 (5)
C35	0.057 (4)	0.046 (4)	0.050 (4)	−0.004 (3)	0.011 (4)	−0.007 (3)
C36	0.057 (4)	0.072 (5)	0.060 (5)	−0.013 (4)	0.018 (4)	−0.006 (4)
C37	0.075 (5)	0.077 (6)	0.063 (6)	−0.010 (4)	0.001 (5)	−0.022 (5)
C38	0.048 (4)	0.053 (4)	0.058 (5)	0.003 (3)	0.018 (4)	−0.004 (4)
C39	0.050 (4)	0.045 (4)	0.050 (4)	−0.002 (3)	0.015 (3)	0.003 (3)
C40	0.052 (4)	0.074 (5)	0.071 (6)	−0.003 (4)	0.024 (4)	−0.016 (4)
C41	0.077 (5)	0.088 (6)	0.061 (6)	−0.002 (4)	0.034 (5)	−0.011 (5)
C42	0.074 (5)	0.065 (5)	0.042 (4)	−0.003 (4)	0.013 (4)	0.005 (4)
C43	0.060 (4)	0.054 (4)	0.040 (4)	−0.007 (3)	0.009 (3)	0.002 (3)
C44	0.053 (4)	0.046 (4)	0.046 (4)	−0.002 (3)	0.013 (3)	0.006 (3)
C45	0.078 (6)	0.133 (8)	0.054 (5)	−0.017 (5)	0.001 (4)	0.025 (5)
C46	0.045 (4)	0.064 (5)	0.066 (5)	−0.010 (3)	0.012 (4)	−0.007 (4)
C47	0.079 (6)	0.102 (7)	0.080 (7)	0.013 (5)	0.007 (5)	−0.003 (5)
C48	0.068 (4)	0.064 (5)	0.043 (4)	0.012 (4)	0.018 (4)	−0.003 (4)
N1	0.062 (4)	0.050 (4)	0.074 (5)	0.010 (3)	0.016 (4)	0.003 (4)
N2	0.044 (3)	0.047 (3)	0.062 (4)	0.005 (3)	0.014 (3)	0.003 (3)
N3	0.044 (3)	0.052 (3)	0.052 (4)	0.000 (2)	0.010 (3)	−0.006 (3)
S1	0.0686 (13)	0.0964 (17)	0.145 (2)	−0.0332 (12)	0.0477 (15)	−0.0558 (17)
S2	0.094 (2)	0.142 (3)	0.161 (3)	0.0396 (18)	−0.038 (2)	−0.016 (2)
S3	0.158 (3)	0.122 (2)	0.0708 (17)	0.066 (2)	0.0515 (17)	0.0365 (16)
O1	0.073 (3)	0.082 (4)	0.059 (4)	−0.009 (3)	−0.002 (3)	0.003 (3)
O2	0.059 (3)	0.061 (3)	0.044 (3)	0.010 (2)	0.007 (2)	−0.003 (2)
O3	0.066 (3)	0.048 (3)	0.089 (4)	0.009 (2)	0.021 (3)	0.005 (3)
O4	0.051 (3)	0.046 (3)	0.084 (4)	−0.001 (2)	0.023 (3)	−0.002 (3)
O5	0.060 (3)	0.084 (4)	0.043 (3)	−0.012 (2)	0.005 (2)	0.006 (3)
O6	0.048 (2)	0.069 (3)	0.046 (3)	−0.002 (2)	0.015 (2)	−0.009 (2)

### Geometric parameters (Å, °)

**Table:**

Nd—O4	2.375 (4)	C23—H23A	0.9300
Nd—O6	2.388 (4)	C24—C25	1.421 (10)
Nd—O2	2.393 (4)	C24—C29	1.423 (9)
Nd—N5	2.489 (8)	C25—C26	1.336 (12)
Nd—N6	2.517 (7)	C25—H25A	0.9300
Nd—N4	2.530 (7)	C26—C27	1.385 (12)
Nd—O1	2.742 (5)	C26—H26A	0.9300
Nd—O5	2.778 (5)	C27—C28	1.359 (10)
Nd—O3	2.843 (5)	C27—H27A	0.9300
C1—C2	1.530 (12)	C28—O3	1.357 (8)
C1—H1B	0.9600	C28—C29	1.400 (9)
C1—H1C	0.9600	C29—O4	1.302 (7)
C1—H1D	0.9600	C30—O3	1.417 (8)
C2—C3	1.366 (12)	C30—H30A	0.9600
C2—C7	1.397 (12)	C30—H30B	0.9600
C3—C4	1.376 (12)	C30—H30C	0.9600
C3—H3B	0.9300	C31—C32	1.518 (10)
C4—C5	1.380 (10)	C31—H31A	0.9600
C4—H4A	0.9300	C31—H31B	0.9600
C5—C6	1.379 (10)	C31—H31C	0.9600
C5—N1	1.405 (9)	C32—C37	1.369 (10)
C6—C7	1.363 (10)	C32—C33	1.380 (11)
C6—H6A	0.9300	C33—C34	1.379 (10)
C7—H7A	0.9300	C33—H33A	0.9300
C8—N1	1.289 (9)	C34—C35	1.350 (9)
C8—C9	1.413 (10)	C34—H34A	0.9300
C8—H8A	0.9300	C35—C36	1.379 (9)
C9—C14	1.403 (10)	C35—N3	1.427 (8)
C9—C10	1.435 (11)	C36—C37	1.388 (9)
C10—C11	1.358 (13)	C36—H36A	0.9300
C10—H10A	0.9300	C37—H37A	0.9300
C11—C12	1.380 (13)	C38—N3	1.311 (7)
C11—H11A	0.9300	C38—C39	1.400 (9)
C12—C13	1.373 (11)	C38—H38A	0.9300
C12—H12A	0.9300	C39—C40	1.409 (9)
C13—O1	1.358 (9)	C39—C44	1.410 (8)
C13—C14	1.412 (10)	C40—C41	1.347 (10)
C14—O2	1.307 (8)	C40—H40A	0.9300
C15—O1	1.450 (8)	C41—C42	1.403 (10)
C15—H15A	0.9600	C41—H41A	0.9300
C15—H15B	0.9600	C42—C43	1.369 (9)
C15—H15C	0.9600	C42—H42A	0.9300
C16—C17	1.503 (10)	C43—O5	1.371 (8)
C16—H16A	0.9600	C43—C44	1.403 (9)
C16—H16B	0.9600	C44—O6	1.301 (7)
C16—H16C	0.9600	C45—O5	1.433 (8)
C17—C18	1.374 (10)	C45—H45A	0.9600
C17—C22	1.380 (10)	C45—H45B	0.9600
C18—C19	1.391 (10)	C45—H45C	0.9600
C18—H18A	0.9300	C46—N4	1.150 (8)
C19—C20	1.355 (9)	C46—S1	1.630 (8)
C19—H19A	0.9300	C47—N5	1.171 (10)
C20—C21	1.379 (9)	C47—S2	1.594 (9)
C20—N2	1.426 (8)	C48—N6	1.145 (8)
C21—C22	1.379 (10)	C48—S3	1.617 (8)
C21—H21A	0.9300	N1—H1A	0.8600
C22—H22A	0.9300	N2—H2A	0.8600
C23—N2	1.284 (8)	N3—H3A	0.8600
C23—C24	1.408 (9)		
			
O4—Nd—O6	143.38 (15)	C21—C22—C17	122.1 (7)
O4—Nd—O2	133.12 (15)	C21—C22—H22A	119.0
O6—Nd—O2	74.67 (15)	C17—C22—H22A	119.0
O4—Nd—N5	73.0 (2)	N2—C23—C24	123.4 (6)
O6—Nd—N5	110.2 (2)	N2—C23—H23A	118.3
O2—Nd—N5	129.1 (2)	C24—C23—H23A	118.3
O4—Nd—N6	86.67 (18)	C23—C24—C25	120.9 (7)
O6—Nd—N6	79.11 (18)	C23—C24—C29	120.9 (6)
O2—Nd—N6	73.77 (18)	C25—C24—C29	118.2 (7)
N5—Nd—N6	156.4 (2)	C26—C25—C24	120.3 (8)
O4—Nd—N4	73.90 (18)	C26—C25—H25A	119.8
O6—Nd—N4	70.59 (17)	C24—C25—H25A	119.8
O2—Nd—N4	139.96 (18)	C25—C26—C27	120.6 (8)
N5—Nd—N4	82.2 (2)	C25—C26—H26A	119.7
N6—Nd—N4	80.6 (2)	C27—C26—H26A	119.7
O4—Nd—O1	98.57 (16)	C28—C27—C26	122.4 (8)
O6—Nd—O1	117.78 (15)	C28—C27—H27A	118.8
O2—Nd—O1	59.79 (16)	C26—C27—H27A	118.8
N5—Nd—O1	75.5 (2)	O3—C28—C27	128.3 (7)
N6—Nd—O1	120.39 (18)	O3—C28—C29	113.2 (6)
N4—Nd—O1	157.76 (19)	C27—C28—C29	118.5 (7)
O4—Nd—O5	142.12 (16)	O4—C29—C28	119.4 (6)
O6—Nd—O5	59.78 (14)	O4—C29—C24	120.7 (6)
O2—Nd—O5	70.97 (15)	C28—C29—C24	119.9 (6)
N5—Nd—O5	69.7 (2)	O3—C30—H30A	109.5
N6—Nd—O5	131.17 (17)	O3—C30—H30B	109.5
N4—Nd—O5	106.50 (18)	H30A—C30—H30B	109.5
O1—Nd—O5	66.39 (15)	O3—C30—H30C	109.5
O4—Nd—O3	58.38 (13)	H30A—C30—H30C	109.5
O6—Nd—O3	142.73 (15)	H30B—C30—H30C	109.5
O2—Nd—O3	75.00 (15)	C32—C31—H31A	109.5
N5—Nd—O3	105.6 (2)	C32—C31—H31B	109.5
N6—Nd—O3	72.00 (18)	H31A—C31—H31B	109.5
N4—Nd—O3	125.27 (17)	C32—C31—H31C	109.5
O1—Nd—O3	61.95 (15)	H31A—C31—H31C	109.5
O5—Nd—O3	127.32 (14)	H31B—C31—H31C	109.5
C2—C1—H1B	109.5	C37—C32—C33	118.4 (8)
C2—C1—H1C	109.5	C37—C32—C31	119.6 (8)
H1B—C1—H1C	109.5	C33—C32—C31	122.0 (9)
C2—C1—H1D	109.5	C34—C33—C32	121.2 (8)
H1B—C1—H1D	109.5	C34—C33—H33A	119.4
H1C—C1—H1D	109.5	C32—C33—H33A	119.4
C3—C2—C7	117.3 (9)	C35—C34—C33	119.8 (8)
C3—C2—C1	122.1 (10)	C35—C34—H34A	120.1
C7—C2—C1	120.7 (11)	C33—C34—H34A	120.1
C2—C3—C4	122.3 (9)	C34—C35—C36	120.6 (7)
C2—C3—H3B	118.8	C34—C35—N3	118.6 (6)
C4—C3—H3B	118.8	C36—C35—N3	120.8 (6)
C3—C4—C5	119.2 (9)	C35—C36—C37	119.3 (7)
C3—C4—H4A	120.4	C35—C36—H36A	120.3
C5—C4—H4A	120.4	C37—C36—H36A	120.3
C6—C5—C4	119.8 (8)	C32—C37—C36	120.8 (7)
C6—C5—N1	117.6 (7)	C32—C37—H37A	119.6
C4—C5—N1	122.5 (8)	C36—C37—H37A	119.6
C7—C6—C5	119.8 (8)	N3—C38—C39	123.9 (6)
C7—C6—H6A	120.1	N3—C38—H38A	118.0
C5—C6—H6A	120.1	C39—C38—H38A	118.0
C6—C7—C2	121.6 (9)	C38—C39—C40	119.9 (6)
C6—C7—H7A	119.2	C38—C39—C44	120.4 (6)
C2—C7—H7A	119.2	C40—C39—C44	119.7 (6)
N1—C8—C9	122.6 (7)	C41—C40—C39	121.1 (7)
N1—C8—H8A	118.7	C41—C40—H40A	119.5
C9—C8—H8A	118.7	C39—C40—H40A	119.5
C14—C9—C8	120.7 (7)	C40—C41—C42	120.3 (7)
C14—C9—C10	118.0 (8)	C40—C41—H41A	119.9
C8—C9—C10	121.3 (8)	C42—C41—H41A	119.9
C11—C10—C9	120.7 (9)	C43—C42—C41	119.4 (7)
C11—C10—H10A	119.6	C43—C42—H42A	120.3
C9—C10—H10A	119.6	C41—C42—H42A	120.3
C10—C11—C12	120.7 (9)	C42—C43—O5	125.0 (6)
C10—C11—H11A	119.7	C42—C43—C44	122.2 (6)
C12—C11—H11A	119.7	O5—C43—C44	112.8 (6)
C13—C12—C11	120.8 (10)	O6—C44—C43	120.4 (6)
C13—C12—H12A	119.6	O6—C44—C39	122.2 (6)
C11—C12—H12A	119.6	C43—C44—C39	117.4 (6)
O1—C13—C12	126.4 (8)	O5—C45—H45A	109.5
O1—C13—C14	113.4 (7)	O5—C45—H45B	109.5
C12—C13—C14	120.1 (8)	H45A—C45—H45B	109.5
O2—C14—C9	122.0 (7)	O5—C45—H45C	109.5
O2—C14—C13	118.4 (7)	H45A—C45—H45C	109.5
C9—C14—C13	119.6 (7)	H45B—C45—H45C	109.5
O1—C15—H15A	109.5	N4—C46—S1	177.7 (7)
O1—C15—H15B	109.5	N5—C47—S2	179.4 (11)
H15A—C15—H15B	109.5	N6—C48—S3	179.1 (7)
O1—C15—H15C	109.5	C8—N1—C5	128.6 (7)
H15A—C15—H15C	109.5	C8—N1—H1A	115.7
H15B—C15—H15C	109.5	C5—N1—H1A	115.7
C17—C16—H16A	109.5	C23—N2—C20	128.4 (6)
C17—C16—H16B	109.5	C23—N2—H2A	115.8
H16A—C16—H16B	109.5	C20—N2—H2A	115.8
C17—C16—H16C	109.5	C38—N3—C35	128.9 (6)
H16A—C16—H16C	109.5	C38—N3—H3A	115.6
H16B—C16—H16C	109.5	C35—N3—H3A	115.6
C18—C17—C22	117.5 (7)	C46—N4—Nd	169.5 (6)
C18—C17—C16	121.3 (7)	C47—N5—Nd	145.5 (7)
C22—C17—C16	121.3 (7)	C48—N6—Nd	156.7 (6)
C17—C18—C19	121.4 (7)	C13—O1—C15	118.2 (7)
C17—C18—H18A	119.3	C13—O1—Nd	115.5 (4)
C19—C18—H18A	119.3	C15—O1—Nd	126.1 (5)
C20—C19—C18	119.5 (7)	C14—O2—Nd	127.0 (4)
C20—C19—H19A	120.2	C28—O3—C30	118.4 (6)
C18—C19—H19A	120.2	C28—O3—Nd	114.2 (4)
C19—C20—C21	120.8 (7)	C30—O3—Nd	127.1 (4)
C19—C20—N2	121.5 (6)	C29—O4—Nd	130.7 (4)
C21—C20—N2	117.6 (6)	C43—O5—C45	117.5 (6)
C20—C21—C22	118.7 (7)	C43—O5—Nd	113.7 (4)
C20—C21—H21A	120.6	C45—O5—Nd	128.6 (4)
C22—C21—H21A	120.6	C44—O6—Nd	126.7 (4)
			
C7—C2—C3—C4	0.6 (14)	O5—Nd—N5—C47	131.5 (13)
C1—C2—C3—C4	−178.2 (9)	O3—Nd—N5—C47	6.9 (13)
C2—C3—C4—C5	−0.5 (14)	O4—Nd—N6—C48	93.5 (14)
C3—C4—C5—C6	0.1 (12)	O6—Nd—N6—C48	−120.4 (14)
C3—C4—C5—N1	177.5 (7)	O2—Nd—N6—C48	−43.4 (14)
C4—C5—C6—C7	0.0 (11)	N5—Nd—N6—C48	123.6 (14)
N1—C5—C6—C7	−177.5 (7)	N4—Nd—N6—C48	167.7 (14)
C5—C6—C7—C2	0.2 (12)	O1—Nd—N6—C48	−4.5 (15)
C3—C2—C7—C6	−0.5 (13)	O5—Nd—N6—C48	−88.5 (14)
C1—C2—C7—C6	178.4 (8)	O3—Nd—N6—C48	35.7 (14)
N1—C8—C9—C14	0.5 (12)	C12—C13—O1—C15	11.3 (12)
N1—C8—C9—C10	179.5 (8)	C14—C13—O1—C15	−170.5 (7)
C14—C9—C10—C11	0.1 (13)	C12—C13—O1—Nd	−163.8 (7)
C8—C9—C10—C11	−178.9 (9)	C14—C13—O1—Nd	14.4 (8)
C9—C10—C11—C12	0.8 (16)	O4—Nd—O1—C13	−153.4 (5)
C10—C11—C12—C13	−0.5 (16)	O6—Nd—O1—C13	31.3 (5)
C11—C12—C13—O1	177.3 (9)	O2—Nd—O1—C13	−18.0 (4)
C11—C12—C13—C14	−0.8 (13)	N5—Nd—O1—C13	136.8 (5)
C8—C9—C14—O2	−2.0 (11)	N6—Nd—O1—C13	−62.2 (5)
C10—C9—C14—O2	179.0 (7)	N4—Nd—O1—C13	138.5 (6)
C8—C9—C14—C13	177.7 (7)	O5—Nd—O1—C13	63.0 (5)
C10—C9—C14—C13	−1.3 (11)	O3—Nd—O1—C13	−106.3 (5)
O1—C13—C14—O2	3.0 (10)	O4—Nd—O1—C15	32.0 (6)
C12—C13—C14—O2	−178.7 (7)	O6—Nd—O1—C15	−143.3 (6)
O1—C13—C14—C9	−176.6 (7)	O2—Nd—O1—C15	167.4 (6)
C12—C13—C14—C9	1.7 (11)	N5—Nd—O1—C15	−37.8 (6)
C22—C17—C18—C19	−0.4 (12)	N6—Nd—O1—C15	123.2 (6)
C16—C17—C18—C19	−179.8 (7)	N4—Nd—O1—C15	−36.1 (8)
C17—C18—C19—C20	−0.3 (12)	O5—Nd—O1—C15	−111.6 (6)
C18—C19—C20—C21	1.8 (11)	O3—Nd—O1—C15	79.1 (6)
C18—C19—C20—N2	178.8 (6)	C9—C14—O2—Nd	155.3 (5)
C19—C20—C21—C22	−2.5 (11)	C13—C14—O2—Nd	−24.4 (9)
N2—C20—C21—C22	−179.6 (6)	O4—Nd—O2—C14	94.2 (6)
C20—C21—C22—C17	1.8 (12)	O6—Nd—O2—C14	−113.8 (5)
C18—C17—C22—C21	−0.4 (12)	N5—Nd—O2—C14	−10.0 (6)
C16—C17—C22—C21	179.0 (7)	N6—Nd—O2—C14	163.3 (6)
N2—C23—C24—C25	−177.4 (8)	N4—Nd—O2—C14	−144.3 (5)
N2—C23—C24—C29	3.1 (12)	O1—Nd—O2—C14	22.2 (5)
C23—C24—C25—C26	177.8 (9)	O5—Nd—O2—C14	−51.0 (5)
C29—C24—C25—C26	−2.7 (14)	O3—Nd—O2—C14	88.1 (5)
C24—C25—C26—C27	1.8 (16)	C27—C28—O3—C30	−9.3 (12)
C25—C26—C27—C28	0.1 (16)	C29—C28—O3—C30	170.8 (7)
C26—C27—C28—O3	179.2 (9)	C27—C28—O3—Nd	165.9 (7)
C26—C27—C28—C29	−0.9 (14)	C29—C28—O3—Nd	−14.0 (8)
O3—C28—C29—O4	0.8 (10)	O4—Nd—O3—C28	15.7 (5)
C27—C28—C29—O4	−179.1 (7)	O6—Nd—O3—C28	154.0 (4)
O3—C28—C29—C24	179.9 (6)	O2—Nd—O3—C28	−169.5 (5)
C27—C28—C29—C24	0.0 (11)	N5—Nd—O3—C28	−42.4 (5)
C23—C24—C29—O4	0.4 (11)	N6—Nd—O3—C28	113.0 (5)
C25—C24—C29—O4	−179.1 (7)	N4—Nd—O3—C28	49.1 (5)
C23—C24—C29—C28	−178.7 (7)	O1—Nd—O3—C28	−106.1 (5)
C25—C24—C29—C28	1.8 (11)	O5—Nd—O3—C28	−118.4 (5)
C37—C32—C33—C34	−1.6 (14)	O4—Nd—O3—C30	−169.6 (7)
C31—C32—C33—C34	−179.1 (9)	O6—Nd—O3—C30	−31.3 (7)
C32—C33—C34—C35	1.7 (13)	O2—Nd—O3—C30	5.2 (6)
C33—C34—C35—C36	−0.9 (12)	N5—Nd—O3—C30	132.3 (6)
C33—C34—C35—N3	176.1 (7)	N6—Nd—O3—C30	−72.3 (6)
C34—C35—C36—C37	0.0 (11)	N4—Nd—O3—C30	−136.2 (6)
N3—C35—C36—C37	−176.9 (7)	O1—Nd—O3—C30	68.6 (6)
C33—C32—C37—C36	0.8 (13)	O5—Nd—O3—C30	56.2 (6)
C31—C32—C37—C36	178.4 (8)	C28—C29—O4—Nd	18.9 (9)
C35—C36—C37—C32	0.0 (12)	C24—C29—O4—Nd	−160.2 (5)
N3—C38—C39—C40	178.8 (6)	O6—Nd—O4—C29	−155.9 (5)
N3—C38—C39—C44	−2.8 (10)	O2—Nd—O4—C29	−25.1 (7)
C38—C39—C40—C41	178.9 (7)	N5—Nd—O4—C29	103.0 (6)
C44—C39—C40—C41	0.5 (11)	N6—Nd—O4—C29	−89.1 (6)
C39—C40—C41—C42	0.7 (12)	N4—Nd—O4—C29	−170.3 (6)
C40—C41—C42—C43	−2.1 (12)	O1—Nd—O4—C29	31.1 (6)
C41—C42—C43—O5	179.5 (7)	O5—Nd—O4—C29	93.3 (6)
C41—C42—C43—C44	2.2 (11)	O3—Nd—O4—C29	−18.3 (5)
C42—C43—C44—O6	179.3 (6)	C42—C43—O5—C45	−11.0 (10)
O5—C43—C44—O6	1.7 (9)	C44—C43—O5—C45	166.5 (6)
C42—C43—C44—C39	−1.0 (10)	C42—C43—O5—Nd	163.6 (6)
O5—C43—C44—C39	−178.6 (6)	C44—C43—O5—Nd	−18.9 (7)
C38—C39—C44—O6	0.9 (10)	O4—Nd—O5—C43	160.4 (4)
C40—C39—C44—O6	179.4 (6)	O6—Nd—O5—C43	20.6 (4)
C38—C39—C44—C43	−178.8 (6)	O2—Nd—O5—C43	−62.4 (4)
C40—C39—C44—C43	−0.3 (10)	N5—Nd—O5—C43	150.5 (5)
C9—C8—N1—C5	−173.3 (7)	N6—Nd—O5—C43	−16.3 (5)
C6—C5—N1—C8	−160.0 (7)	N4—Nd—O5—C43	75.6 (5)
C4—C5—N1—C8	22.5 (11)	O1—Nd—O5—C43	−126.9 (5)
C24—C23—N2—C20	178.3 (7)	O3—Nd—O5—C43	−115.0 (4)
C19—C20—N2—C23	10.6 (11)	O4—Nd—O5—C45	−25.8 (7)
C21—C20—N2—C23	−172.3 (7)	O6—Nd—O5—C45	−165.6 (7)
C39—C38—N3—C35	178.2 (6)	O2—Nd—O5—C45	111.5 (6)
C34—C35—N3—C38	−164.5 (7)	N5—Nd—O5—C45	−35.7 (6)
C36—C35—N3—C38	12.5 (10)	N6—Nd—O5—C45	157.5 (6)
O4—Nd—N4—C46	150 (3)	N4—Nd—O5—C45	−110.6 (6)
O6—Nd—N4—C46	−21 (3)	O1—Nd—O5—C45	46.9 (6)
O2—Nd—N4—C46	10 (4)	O3—Nd—O5—C45	58.8 (7)
N5—Nd—N4—C46	−136 (3)	C43—C44—O6—Nd	22.3 (9)
N6—Nd—N4—C46	61 (3)	C39—C44—O6—Nd	−157.3 (5)
O1—Nd—N4—C46	−137 (3)	O4—Nd—O6—C44	−160.7 (5)
O5—Nd—N4—C46	−70 (3)	O2—Nd—O6—C44	54.3 (5)
O3—Nd—N4—C46	121 (3)	N5—Nd—O6—C44	−72.3 (5)
O4—Nd—N5—C47	−42.1 (12)	N6—Nd—O6—C44	130.2 (5)
O6—Nd—N5—C47	176.5 (12)	N4—Nd—O6—C44	−146.0 (5)
O2—Nd—N5—C47	90.1 (13)	O1—Nd—O6—C44	11.5 (5)
N6—Nd—N5—C47	−73.7 (15)	O5—Nd—O6—C44	−22.3 (5)
N4—Nd—N5—C47	−117.6 (13)	O3—Nd—O6—C44	90.8 (5)
O1—Nd—N5—C47	61.8 (13)		

### Hydrogen-bond geometry (Å, °)

**Table:**

*D*—H···*A*	*D*—H	H···*A*	*D*···*A*	*D*—H···*A*
N1—H1A···O2	0.86	1.86	2.566 (7)	138
N2—H2A···O4	0.86	1.87	2.576 (6)	138
N3—H3A···O6	0.86	1.89	2.589 (7)	137
C38—H38A···S1^i^	0.93	2.72	3.633 (6)	166

Symmetry codes: (i) −*x*+3, −*y*+1, −*z*+3.
